# Monocytes across life span in HIV infection: lights and shadows

**DOI:** 10.1097/COH.0000000000000910

**Published:** 2025-01-08

**Authors:** Alessia Neri, Giulio Olivieri, Chiara Pighi, Donato Amodio, Nicola Cotugno, Paolo Palma

**Affiliations:** a Clinical and Research Unit of Clinical Immunology and Vaccinology, Bambino Gesù Children's Hospital, IRCCS; b PhD Program in Immunology, Molecular Medicine and Applied Biotechnology; c Chair of Pediatrics, Department of Systems Medicine, University of Rome “Tor Vergata” Roma, Italy

**Keywords:** HIV, inflammation, long-term reservoir, monocyte subsets, monocytes

## Abstract

**Purpose of review:**

This review highlights the role of monocytes in the pathogenesis of HIV-1 infection, focusing on their involvement in the inflammatory response and their function as viral targets and long-term reservoirs.

**Recent findings:**

Monocytes have been categorized into three subsets: classical, intermediate, and nonclassical, each with distinct functional characteristics. Advances in genetic sequencing technologies have enabled a more in-depth exploration of the phenotypic and functional variations among these subsets, particularly in the context of HIV. These findings underscore their role as crucial components of the immune response and as reservoirs for the virus.

**Summary:**

Previous studies on the role of monocytes have demonstrated their contribution to persistent infection and chronic immune activation, especially in adults living with HIV. The lessons learned from these studies should now be harnessed to design studies focused on newborns and children with vertically acquired HIV.

## INTRODUCTION

Cells of the myeloid lineage play an important role in the innate response against HIV-1 and subsequently throughout the course of the disease [1]. Indeed, the induction of the innate immune response can induce host restriction factors that suppress the replication and spread of HIV-1, activating immune response towards viral control [2]. Monocytes (MO) respond to various signals guiding macrophage (Mϕ) differentiation, polarization, and functioning. MO/Mϕ both detect and eliminate pathogens, function as antigen-presenting cells (APC) to initiate the adaptive immune response, and play roles in both pro- and anti-inflammatory reactions, contributing to tissue repair [1]. This narrative review focuses on the overall role of MO in the pathogenesis of HIV-1 infection by serving as viral targets and reservoirs, contributing to the outcomes of HIV infection. 

**Box 1:**
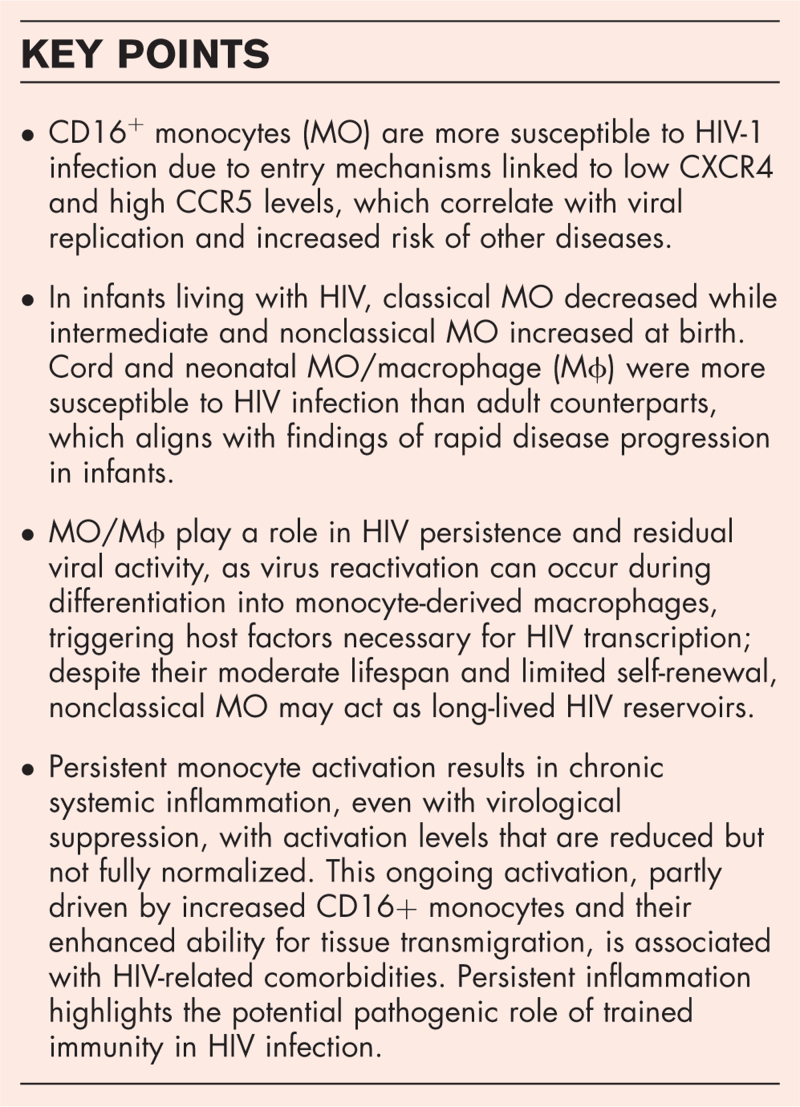
no caption available

## MONOCYTES AND FUNCTIONAL DIFFERENCES ACROSS SUBSETS

**FIGURE 1:**
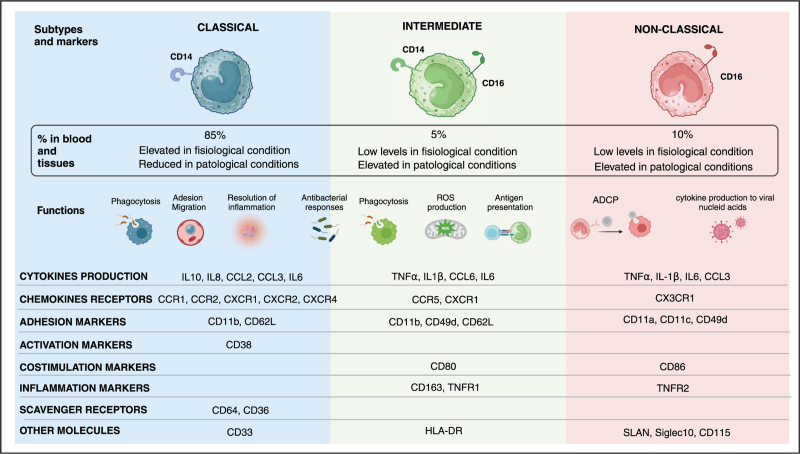
Classification and characterization of monocyte subsets. The figure illustrates the three main subsets of human monocytes: classical (CD14^+^CD16^−^), intermediate (CD14^+^CD16^+^), and nonclassical (CD14^−^CD16^+^). The figure presents the relative proportions of each subset in peripheral blood under both physiological and pathological conditions. It highlights the functions of each subset, along with the expression of key markers and cytokines, emphasizing the subset that exhibits the highest expression levels for each marker.

MO originate from CD34^+^ myeloid progenitor cells, circulate in the bloodstream and lymph for about 70 h, and then migrate into tissues where they differentiate into monocyte-derived macrophages (MDM). MO are crucial for maintaining tissue homeostasis and detecting pathogens through Toll-like receptors (TLRs) and other pattern recognition receptors (PRRs) and presenting antigens [3–8]. MO are divided in three phenotypically and functionally distinguished subpopulations and their distribution changes in pathological states, playing a distinct role in the different contexts [9–11]. Currently, the three subsets are designated through the expression of the CD14 (LPS receptor) and CD16 (FcγRIIIA) markers: classical (CL, CD14^++^CD16−), intermediate (ITM; CD14^+^CD16^+^), and non-classical (NC; CD14^−^CD16^++^) [12] (Fig. 1). Cros *et al.* were the first to compare gene expression by different subsets, purified from healthy controls (HC) samples [5]. Recent advancements identified additional surface markers such as CCR2, CX3CR1, HLA-DR, CD86, CD64 and CD33, which further refine this classification. These markers are particularly helpful in the distinction between ITM and NC MO [5,13,14]. Indeed, distinguishing NC (CD14low) from ITM MO (CD14high) subsets based on CD14 gradual variation of its expression is challenging, due also to the variability under *in vitro* conditions. Furthermore, SLAN has emerged as a specific candidate marker for defining NC MO [15].

Under physiological conditions, MO originate from the bone marrow as CL MO [16], which constitute 80–90% of circulating MO. CL MO migrate into tissues and differentiate into MDM and are highly phagocytic, expressing high levels of scavenger receptors, such as CD64 and CD36, in contrast to the other two subsets. They also express CCR2 and CD11b, enhancing their migration to inflamed tissues. CL MO play a key role in resolving inflammation and produce GM-CSF and interleukin (IL)-10 in response to pathogen associated molecular patterns (PAMPs) and damage associated molecular patterns (DAMPs) stimulation, underscoring their involvement in innate immune responses, tissue remodeling and demonstrating to be less inflammatory than their counterparts [5,17].

Tak *et al.* investigated the dynamics of human MO circulation, proposing that fewer than 10% of CL MO mature into the ITM subset, with 82–86% of these ITM MO later differentiating into circulating NC MO [18]. Both ITM and NC MO are considered inflammatory subsets. ITM MO demonstrate elevated expression levels of HLA-DR, CD80, CD86, CD163, TNFR1 and CCR5, indicating roles in antigen presentation and inflammation [19–22]. They are the main producers of tumor necrosis factor alpha (TNFα), IL-1β and IL-6 [5]. Notably, ITM MO generate the highest baseline reactive oxygen species (ROS) production [23], further linking them to inflammation and bacterial cues [5].

On the other hand, NC MO exhibit high expression of SLAN, CD115, siglec10, CD86, and TNFR2, underscoring their pivotal role in inflammation [9,20,24]. They also express CX3CR1 and CD11c, enhancing their potential to migrate to the vessel wall and “*patrolling*”, where they help in debris removal [5,25,26]. NC MO perform antibody-dependent phagocytosis via CD16, an Fc receptor for IgG antibodies [11]. While unresponsive to bacterial signals, they produce pro-inflammatory cytokines like TNFα, IL-1β, and CCL3 following viral nucleic acids and immune complexes stimuli [5] (Table 1).

**Table 1:** Expression of several markers in different monocyte subsets

Markers	Classical	Intermediate	Nonclassical
CD14 [12]	++	+	−
CD16 [12]	−	+	++
CD64 [23]	++	+	+
CD86 [24]	+	+	++
CD33 [17]	++	ND	+
HLA-DR [39]	+	++	+
CCR1 [17,18]	++	+	−
CCR2 [17,18]	++	+	−
CCR5 [18]	+	++	+
CXCR1 [17,18]	+	+	−
CXCR2 [17]	+	−	−
CXCR4 [17]	+	ND	ND
CX3CR1 [5]	+	+	++
CD80 [17]	ND	+	ND
SLAN [9]	ND	ND	+
Siglec10 [17]	−	ND	+
CD115 [9]	ND	ND	+
CD163 [3,145]	+	++	ND
TNFR1 [145]	+	++	−
TNFR2 [145]	−	+	++
CD11a [23]	ND	ND	+
CD11b [5,146,147^▪^]	++	++	+
CD11c [147^▪^]	+	+	++
CD62L [39,147^▪^]	+	+	−
CD38 [39]	++	+	+
CD49d [24,148^▪▪^]	ND	+	+
CCL2 [5,22]	++	+	+
CCL3 [5,22]	+	ND	+
TNFα [17,149]	+	++	++
IL-1β [5]	ND	++	++
IL-8 [5,9]	++	+	+
IL-10 [5,24]	++	+	+
IL-6 [5,9]	+	+	+
ROS production [17,150]	+	++	−

This table summarizes the expression levels of key markers that are commonly reported in the three monocyte subsets. The expression levels are indicated as high (++), moderate (+), not detected (−) or no data available (ND) based on available data. TNFα, tumor necrosis factor alpha; IL, interleukin.

The molecules expressed and produced by different subsets are not consistently reported across various studies in literature, particularly given the similarity in the characteristics of intermediates with both classic and nonclassical phenotypes.

## PHYSIOLOGICAL ROLE OF MONOCYTES ACROSS AGE: FROM CHILDHOOD TO ADULTHOOD

**FIGURE 2:**
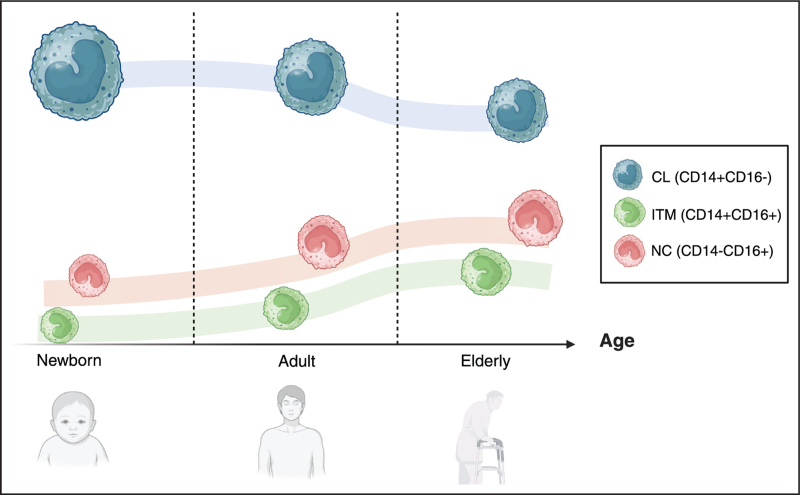
Representative trends of monocyte subsets across different ages: neonates, adults, and the elderly. The size of the monocyte reflects the percentage of a specific subset in relation to the other monocyte subsets. At birth and during infancy, classical monocytes constitute nearly the entirety of the monocyte population (around 90%). With age, there is a gradual reduction in this subset, although it remains the predominant group, in favor of an increase in inflammatory subsets. CL, classical; ITM, intermediate; NC, nonclassical.

Investigating MO population changes across different ages is essential to understand their role during infections. Newborns have different MO frequencies in blood compared to later ages and distinct subsets distribution and turnover rates are reported in animal models [27]. Indeed, MO may represent 7–38% of all circulating mononuclear cells in cord blood of term infants [28,29]. Neonates and infant macaques showed high MO turnover, reaching adult levels approximately at 6 months of age, likely due to MO trafficking to repopulate and differentiate into tissue Mϕ. Moreover, MO may compensate for the immature adaptive immune system that is not developed in the first stages of life, and they may help to control the early infections. Studies also show that MO/Mϕ from cord blood are less mature in phagocytosis, chemotaxis and metabolism compared to adult cells [30–35]. Valiathan *et al.* observed that MO levels were similar across infants, children and adolescents, but significantly decreased in the adults to then rise again in the elderly. MO activation also increased with age although a gender driven difference was underlined as females in adolescence and childhood showed lower MO percentages than males, with reduced cytotoxic activity in young females compared to males [36]. Byrne *et al.* noted higher CCR2 and lower CX3CR1 expression in CL MO in adults, while NC MO showed the opposite pattern. In their study, they found that CD11b, CD11c and CD163 expression in CL MO peaked in mid adult life compared with older control [37]. George *et al.* determined a higher frequency of CD14^+^ CD16^+^ population in middle and old age compared to young in healthy control participants, together with a higher HLA-DR expression on CL and ITM MO in old healthy controls than young healthy controls [38]. Seidler *et al.* reported a positive correlation between age and CD14^+^CD16^+^ population, which are increased in adults (> 30 years) compared to the younger (<30 years). Moreover, the expression of HLA-DR and CX3CR1 was significantly lower on CD14^+^CD16^+^ monocytes of older volunteers [39]. Hearps *et al.* demonstrated an impaired phagocytosis and an increased pro-inflammatory response to LPS in monocytes suggesting their dysregulated function during aging. Monocytes from older individuals contained significantly increased basal levels of TNFα, suggesting persistently higher pro-inflammatory activity of these cells in vivo [40]. These data demonstrate significant changes in MO populations and their surface markers during healthy aging (Fig. 2).

## MONOCYTES ROLE IN HIV INFECTION

In the context of HIV infection, MO/Mϕ inflammatory functions can be dysregulated either by direct or bystander mechanisms. This compromised immune response may lead to HIV reactivation and contribute to residual immune dysfunction and increase the risk of non-AIDS-related diseases. However, their involvement in HIV pathogenesis, in the pediatric setting, is not well established.

During HIV-1 infection, increased activation and differentiation of MO in the peripheral blood is observed [1,41], along with changes in MO subsets levels. In people with HIV-1 (PWH), there is a noticeable expansion of ITM MO and NC MO. These alterations on MO compartment reflect residual immune dysfunction and the risk of non-AIDS-related diseases [42]. Moreover the subsets disruption can persist through all stages of infection, even in antiretroviral therapy (ART)-treated subjects, depending on the levels of viral loads, with an increase in NC population that may represent up to 40% of the circulating MO population [43–45]. Han *et al.* showed an increase of both ITM and NC MO in a cohort of ART-naive individuals with chronic HIV-infection compared to HC. ITM MO, but not NC MO, positively correlated with viral load. After ART administration, ITM MO levels decreased along with viral load but remained higher than in HC. In contrast, NC MO levels did not decrease after 1 year of ART, suggesting these two subsets respond differently to ART and HIV [46].

While CL MO are relatively resistant to HIV-1 infection, ITM and NC MO are more susceptible and can harbor proviral DNA in both untreated and treated PWH [1,47,48]. ITM MO can be infected during trafficking, while NC MO can be infected in bone marrow, spleen and tissue, that are potential HIV sanctuaries [5,49,50].

Interestingly, ITM MO that harbors HIV elevated the expression of specific surface junctional proteins, enhancing their selective advantage in transmigrating across the blood−brain barrier (BBB). This may facilitate the establishment and replenishment of viral reservoirs within the central nervous system [51].

Ellery *et al.* described CD16^+^ MO as more permissive to HIV-1 infection, levels correlated with HIV viral replication and the probability of establishment of other pathologies [47]. NC MO present low levels of CXCR4 and high expression of CCR5, making them more prone to HIV infection [47,52,53], as CCR5 plays a key role in early HIV pathogenesis, particularly in the early stages of infection [54,55]. Additionally, NC MO subset contains an inactive form of APOBEC3G, a gene that blocks HIV reverse transcription. In contrast, the active form of the APOBEC3G, which inhibits HIV, is found in the CL MO [47,56,57]. Although little known, CD4 expression is also present in the MO compartment and is considered an important coreceptor of HIV infection in these cells. The expression of the CD4 marker on MO and its role in HIV infection has been well established for some time [58]. MO express significantly higher levels of CD4 than Mϕ. Lodge *et al.* reported high levels of CD4 expression on THP-1 monocytic cell lines, which decrease due to a post transcriptional mechanism (MicroRNAs-221 and -222) as these cells differentiate into Mϕ. Their work also highlights the critical role of CD4 deregulation in HIV infection, showing that higher CD4 levels are necessary for viral entry and efficient productive infection in an *in vitro* model [59]. Moreover Zhen *et al.* demonstrates that CD4–MHC-II interaction represents a potential mechanism contributing to the heightened susceptibility of circulating MO to HIV infection, persistence within the Mϕ compartment, and the establishment of the HIV reservoir [60].

### Pediatric HIV-1 infection

Perinatal HIV-1 infection often progresses more rapidly than in adults and the role of MO/Mϕ in this context remains poorly understood. Notably, the proportion of CL MO was reduced at birth in infants with HIV infection, while ITM and NC MO were expanded. Similarly, ITM MO were higher in newborn macaques than in adults [27]. The note disruptions in the MO distribution in infants with HIV infection also appeared to normalize at subsequent time points following ART initiation [61].

Sperduto *et al.* investigated the susceptibility of neonatal, cord blood and adult MO/Mϕ to HIV at various stages of differentiation *in vitro* culture. Their finding revealed that neonatal and cord MO/Mϕ exhibited higher susceptibility to HIV infection compared to adult counterparts. This discrepancy in susceptibility was most pronounced in cells infected after 11 days of culture, followed by 7 days and finally by 4 days. Their findings align with previous observations that less differentiated cells were prone to be more productively infected [58]. While IL-1β and TNFα have been involved in modulating this susceptibility, their levels were not significantly different in Sperduto's study, in line with other studies [62–65]. In contrast, an increase in DNA synthesis, indicating cell replication, was noted, suggesting that cell proliferation may be necessary for efficient HIV-1 infection [62].

Moreover, MO turnover is linked to disease progression and reduced CD4^+^ T cell levels [66–70]. Sugimoto *et al.* described that in Simian deficiency virus (SIV) infected macaques, MO turnover peaked during the acute infection in neonates [27] and was associated to an increase in ITM subset. These cells are described with various microbial infections [71] and exhibit proinflammatory properties, such as producing high levels of soluble CD163 and CXCL10 [72–74], making these cells more susceptible to infection. These data are in line with findings showing that newborns’ MO are more susceptible to HIV infection in vitro [62,75] and resulting in a rapid disease progression in newborns compared to adults.

### Monocyte driven persistent immune activation and inflammation in HIV infection

Despite the ART treatment, some individuals still exhibit chronic immune activation and inflammation, involving dysregulated and dysfunctional MO linked to HIV comorbidities [42]. MO activation leads to persistent systemic inflammation even under virological suppression, with activation levels reduced but not normalized [76,77]. MO are important predictors of morbidity and mortality [78], with markers such as soluble CD14 and CD163, and CXCL10, remaining elevated compared to HC also in virologically suppressed people, indicating poor prognosis [77–81]. Elevated IL-6 levels are additional signals of ongoing inflammation and are associated with mortality [82]. This chronic condition is linked to a senescent MO phenotype, also in young PWH, especially in the CD16+ subset, which expands prematurely in PWH and is characterized by shorter telomeres [40] and altered levels of CD64, CX3CR1, CD86, CD91, CD38 and CD40 [83]. George *et al.* studied a cohort of ART-treated participants with HIV infection across different ages and identified subjects expressing high or low levels of CD11b on ITM MO. The CD11b^hi^ subset was associated with baseline inflammatory characteristics, including elevated plasma sTNFR1 and higher baseline CXCL10 gene expression levels, as well as increased TNF and IL-6 gene expression following LPS stimulation. This subpopulation also demonstrated the ability to suppress antigen-specific CD4^+^ T-cell proliferation and exhibited an inverse correlation with serum antibody titers following H1N1 vaccination, demonstrating a contribute of MO in the impairment of flu vaccine responses [38].

### Antibody dependent cellular phagocytosis

Investigating the role of antibody dependent cellular phagocytosis (ADCP) in HIV infection is of crucial importance. Indeed, ADCP may revoke HIV-infected cells, affect immune complex-induced inflammation and enhance adaptive immune response against the virus [84]. MO, along with NK cells responsible for the antibody dependent cellular cytotoxicity (ADCC), are key players in this process. However, peripheral MO from PWH present defective phagocytosis, regardless of viral load levels [85–87]. This impairment may result from various factors, including loss of FcɣR intracellular signaling molecules, dysregulation of actin polymerization involved in pseudopods [85], altered apoptotic responses and alterations in TLR signaling [88]. Using a new ADCP high-throughput assay, Dufast *et al.* found increased in FcɣRI expression on MO during the acute HIV infection, while FcɣRII expressions decreased on phagocytic in chronic and untreated HIV infection. These alterations in FcɣRs expression are linked to a diminished ability to respond to antibody-opsonized targets and impaired MO function due to HIV infection [89].

### Impact of monocyte/macrophage on the viral reservoir

Myeloid cells play a role in the latent reservoir in PWH. In animal models, MO/Mϕ can independently maintain HIV infection, irrespective of CD4^+^ T cells [90]. Similarly, humanized mice models have revealed that HIV can persist in tissue resident Mϕ even during ART-mediated viral suppression [91]. Research on macaques with suppressed SIV infections has demonstrated the existence of myeloid reservoirs capable of replication in both blood and tissues, even after prolonged viral suppression [92]. These findings highlight the role for MO/Mϕ in HIV persistence and residual viral activities. Additionally, HIV reservoirs in Mϕ can rebound after treatment interruption, with HIV DNA detected in highly purified MO [93–96] and Mϕ isolated from various organs, including brain, urogenital tract, lung, liver, gut, and lymph nodes [97–100], that serves as sanctuaries for cells vulnerable to infection [101].

Viral reactivation may occur during differentiation into MDM, as this process can trigger the expression of host factors necessary for the transcription of full-length HIV, such as positive transcription elongation factor b (P-TEFb) or Cyclin T1 (CycT1) [102,103]. While MO typically expresses low levels of CycT1, its upregulation occurs upon differentiation into MDM, potentially triggering HIV reactivation [104].

Blanco *et al.* established an MDM model of HIV latency that revealed differences in latency regulation between MO and MDMs, with latent provirus transitioned to an actively replicating state, posing a potential risk for viral dissemination in tissues [105^▪▪^].

These findings reflect similar variability in HIV reactivation and responses to latency-reversing agents (LRAs) seen in multiple T cell latency models [106,107]. Blanco *et al.* validated these observations using primary human CD14+ MO from various donors, showing that HIV reactivation progressively increased alongside macrophage differentiation, identifying several mechanisms involved in latency reversal, including PKC and nuclear factor (NF)-κB pathways activation in line with previous research reporting HIV reactivation over time in primary HIV-infected MDMs [105^▪▪^,^108^,^109^].

Despite the importance of the myeloid viral reservoir in HIV pathogenesis, research assessing reactivatable reservoirs in MO [94,96] frequently employs assays not tailored to their unique biology, resulting in inconsistent findings. No standardized techniques exist for assessing HIV reactivation from the MO reservoir, and the function of these cells in sustaining Mϕ-induced tissue reservoirs remains unclear. An essential concern in the context of quantifying HIV DNA in MO or Mϕ is the purity of the samples, as reports show significant variability in HIV DNA identification among donors [93,95,96]. MO harboring replicative viruses might replenish tissue-Mϕ reservoirs upon transforming into MDM. Recently, Veenhuis *et al.* developed an MDM quantitative viral outgrowth assay (MDM-QVOA) to measure replicant-competent and DNA MDM reservoirs in virologically suppressed PWH and directly compared them to CD4^+^ T cell reservoirs. All participants had detectable HIV DNA in their MDM although at levels 10 times lower than those in CD4+ T cells with intact HIV genomes in MO capable of infecting CD4^+^ T cells. These findings indicate consistent presence of HIV DNA in MDM, suggesting that MO might constitute a stable HIV reservoir [110^▪▪^]. The major challenges lie in identifying the maturation stages at which these cells become infected. Despite moderate life span and lack self-renewing potential, various theories proposed the possibility of HIV-1 infection in the maturation stage of NC MO or as tissue-resident Mϕ, indicating a potential role of these cells as long-lived HIV reservoirs. Mϕ are distinguished between long-lived tissue-resident Mϕ, with embryonic origins and lifespan ranging from week to decades, and infiltrating Mϕ, which have estimated half-lives. Long-lived Mϕ present the unique ability to self-renew, enabling them to sustain HIV-1 infection and evade the effects of ART by maintaining the virus in a quiescent state. These cells also develop mechanisms to resist apoptosis and sustain the self-renewal capacity, providing an immune sanctuary for latent HIV-1 [111–113]. In support of this theory, some in *vivo* evidence suggests that a limited CD34^+^ myeloid progenitor HIV reservoir exists in certain individuals [114,115], although this was not reported in other studies [116,117]. These findings underscore the possible risk that HIV-infected monocytes and Mϕ present for viral spread and resurgence when ART is interrupted in PWH.

### Innovative role of trained immunity in pediatric HIV infection

The concept of trained immunity, first introduced by Netea [118], refers to the long-term reprogramming of innate immune cells after exposure to external or internal stimuli. This reprogramming enhances the immune system's ability to respond more effectively to subsequent challenges, while still returning to a physiological baseline. The adaptation occurs through epigenetic and metabolic changes, leading to one of two outcomes: either an enhanced immune response to the second stimulus or tolerance, where the second response is diminished or suppressed [119].

Trained immunity results in widespread protection against pathogens but its effect during chronic infection, like HIV infection, is less understood. In HIV context, trained immunity may lead to heightened inflammation due to enhanced innate immune response to TLR stimulation [120]. Some studies reported modifications during the HIV infection that may be related to trained immunity mechanisms. For example, a study from Espindola *et al.* showed that MO from untreated subjects with HIV infection present a dysregulation of phagocytosis after *M. tuberculosis* (Mt) stimulation, along with increased activation and ROS and cytokines production, correlated with immune dysregulation, senescence and inflammaging in this population. These pro-inflammatory characteristics and impaired antituberculosis activity were linked to epigenetic modifications in both untreated and ART-treated subjects with HIV infection, demonstrating that ART reduced but did not fully restore normal function [121]. Specifically, Zhang *et al.* identified three MO subpopulations following the induction of the trained immunity, resulting in enhancement of the inflammatory response and affecting the mechanisms in infection and inflammation [122^▪^]. Dos Santos *et al.* demonstrated an increased response upon stimulation and a transcriptomic profile that is less inflammatory and more antiviral in MO from persistent controllers’ subjects [123]. Additionally, the HIV protein Nef has been defined as the major pathogenic factor of HIV-1 by activating the Akt–mTOR pathway [124–127], a known mechanism in trained immunity. This pathway increased aerobic glycolysis and cholesterol biosynthesis [118], both key to MO activation in individuals with HIV infection. MO from PWH, including those on ART with undetectable viral loads, show higher glucose mechanism elevated cholesterol efflux and biosynthesis, abundance of lipid rafts and epigenetics changes that promote the expression of inflammatory genes mediated by HIV protein Nef impairs [128,129]. Lipid rafts, which stabilize TLR4 signaling after cell activation, become more stable to lead the agonist accommodation and contribute to a long-term permanent maintenance of TLR4 activation [130–132]. Resident Mϕ, derived from circulating MO, support this mechanism despite the short lifespan of MO [133,134]. In addition, trained myeloid progenitor cells in the bone marrow may provide support in the production of trained MO in subjects living with HIV. Interestingly, the HIV envelope glycoprotein gp120, can trigger TLR2 and TLR4 signaling [135], mimicking trained immunity like β-glucan. Additionally, low concentrations of Nef have been detected in the blood of individuals with HIV infection with undetectable viral load [136], perpetuating inflammation [128,129,137], confirmed by IL-1β overexpression, hallmark of trained immunity [138]. This persistent inflammation highlights the potential pathogenic role of trained immunity in HIV infection. In pediatric settings, especially in cases of vertical transmission, the effects of chronic virus exposure and trained immunity mechanisms, pathogen exposure and vaccines are critical during early life when the innate immune system is extremely important. Understanding the role of MO in this context is fundamental, as they could be a potential therapeutic target in pediatric HIV infection.

### Advances in monocyte transcriptomics and metabolism: new insights and discoveries

Recent advances in single cell RNA sequencing have been used to examine transcriptional complexities associated with viral infection, viral latency, and cellular components in with HIV related comorbidities [139,140,141^▪▪^].

Bashore *et al.* presented a large single multimodal dataset of circulating human MO, reevaluating the oversimplified traditional CD14/CD16 classification and identifying two additional development pathways beyond the CL to ITM to NC differentiation. One culminated into an IFN-responsive MO subset, characterized by antiviral gene expression, without definition of surface proteins. Additionally, an ITM subset with high expression levels of MHC related genes and antigen presentation was identified [142]. Wu *et al.* confirmed the role of MHC-I and MHC-II in MO, finding increased MHC-I expression and phagocytosis pathway activation in HIV+ patients with viremia, compared with ones that controlled the virus under detection levels [143].

Leon-Rivera *et al.* suggested that ART might affect MO dysfunction performing a characterization of primary HIV+ and HIVexposure mature MO with and without ART. HIV+ and HIVexposure MO presented differential gene expression, with HIV+ MO showing an increased cell migration with a selective advantage to transmigrate across BBB and decreased apoptosis, reported as prevalent in PWH [144,145]. ART therapy might also modulate the mature MO transcriptome, contributing to viral reservoir and by blocking later stages in viral transcription. Authors identified nine clusters, with only one unique to untreated HIV infection displaying anti-inflammatory phenotype, inhibition of LPS-induced ROS and diminished after ART associated with dysregulation in HIV-associated comorbidities [146,147^▪^]. Blanco *et al.*, using a HIV latency MO model, linked redox protein imbalances to HIV transcription [105^▪▪^], while Guo *et al.* observed increased ITM MO with inflammatory phenotype in the HIV-1-Tb co-infection, with an enhanced migration capacity to BBB involved in the development of cognitive impairment [148^▪▪^].

Moreover, HIV-1 is also known to contribute to mitochondrial dysfunction, even in people on ART. Munoz-Muela *et al.* reported higher levels of GLUT1 in nontreated and immunological nonresponders, as previously described [149]. GLUT-1 is a key inflammation mediator together with mitochondrial disruption, contributing HIV-1 infectivity and replication and is upregulated in MO in PWH, despite the ART [150,151].

## CONCLUSION AND PERSPECTIVE

**FIGURE 3:**
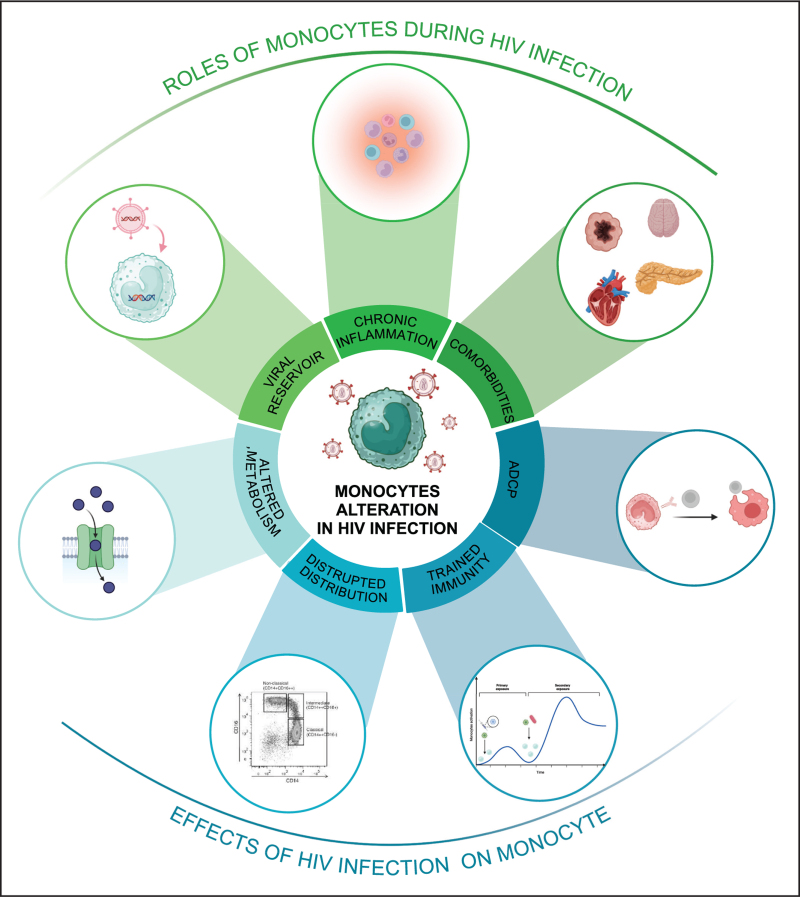
Overview of the effects of HIV infection on monocytes and the role of monocytes in HIV infection. The figure summarizes the bidirectional interactions between HIV infection and monocytes. On one side, it illustrates how HIV infection alters monocytes, on the other side how these altered monocytes impact HIV disease progression.

MO play a complex role in HIV-1 infection, acting as both crucial components of the immune response and reservoirs for the virus, which contributes to persistent infection and chronic immune activation (Fig. 3). In pediatric cases, the developing immune system and distinct characteristics of MO subsets require particular attention, especially due to the rapid disease progression and higher mortality seen in neonatal HIV infection. To improve treatment outcomes, understanding the mechanisms behind monocyte-driven viral persistence and inflammation is essential. Future research should target these pathways to develop better therapeutic strategies tailored to the unique challenges of HIV-1 infection.

## Acknowledgements

*The authors thank Dr Elena Morrocchi and Arianna Rotili for their contribution to the revision of the text. We acknowledge Ilaria Pepponi and Melania Fantini for administrative assistance.*

### Financial support and sponsorship

*This work was supported by the Italian Ministry of Health with “Current Research funds”. Supported by US National Institutes of Health and Hiv Vaccine to Reduce Reservoir In Children and Adolescent Network Epiical (HVRRICANE, U01AI135941). This work was also supported by federal funds from the NIH through the Pediatric Adolescent Virus Elimination Martin Delaney Collaboratory Project Number 1UM1AI164566-01.*

### Conflicts of interest

*The authors declare that there are no conflicts of interest regarding the publication of this review.*

## REFERENCES AND RECOMMENDED READING

Papers of particular interest, published within the annual period of review, have been highlighted as:

▪ of special interest
▪▪ of outstanding interest
